# Prognostic value of platelet to lymphocyte ratio in non-small cell lung cancer: a systematic review and meta-analysis

**DOI:** 10.1038/srep22618

**Published:** 2016-03-04

**Authors:** Hua Zhang, Liuwei Gao, Bin Zhang, Lianmin Zhang, Changli wang

**Affiliations:** 1 Department of Lung Cancer, Tianjin Medical University Cancer Institute and Hospital, National Clinical Research Center for Cancer, Key Laboratory of Cancer Prevention and Therapy, Tianjin Lung Cancer Center.

## Abstract

The prognostic value of the platelet-to-lymphocyte ratio (PLR) in non-small cell lung cancer (NSCLC) remains controversial. We therefore conducted a meta-analysis of published studies to determine the prognostic value of PLR in NSCLC. A systematic search was performed in PubMed, Web of Science and Embase for relevant studies. The data and characteristics of each study were extracted, and the hazard ratio (HR) at a 95% confidence interval (CI) was calculated to estimate the effect. We also performed subgroup and meta-regression analyses. A total of 2,889 patients in 12 studies were enrolled in this meta-analysis, and the pooled HR of 1.492 (95% CI: 1.231–1.807, *P* < 0.001) indicated that patients with an elevated PLR are expected to have a shorter overall survival (OS) after treatment. This meta-analysis indicates that a high PLR might be a predictive factor of poor prognosis in NSCLC. Further large-cohort studies are needed to confirm these findings.

Lung cancer remains the most lethal malignancy and the most common cause of cancer-related death worldwide, accounting for approximately 1.59 million deaths around the world in 2012^1^. Non-small cell lung cancer (NSCLC) accounts for approximately 85% of all lung cancers. Great advances in surgery, chemotherapy and radiotherapy, as well as the emergence of molecular therapy have revolutionized the management of lung cancer patients. Nevertheless, the clinical outcome of lung cancer remains unsatisfactory due to local tumor recurrence and distant metastasis. Thus, the identification of an efficient and reliable marker to obtain additional prognostic information is essential.

Recently, a marker of the systemic inflammatory response (SIR) - the platelet-to-lymphocyte ratio (PLR) - was identified as a useful indicator for predicting the prognosis of various cancers such as breast cancer^2^, epithelial ovarian cancer^3^, pancreatic cancer^4^, colorectal cancer^5^, and gastric cancer^6^. The prognostic value of PLR in NSCLC has also been investigated^7^,^8^,^9^,^10^,^11^,^12^,^13^,^14^,^15^,^16^,^17^,^18^. However, due to variance in study design and sample size, these studies have reported inconsistent results. It is therefore unknown whether PLR is a suitable prognostic indicator of NSCLC. In this study, we searched for available studies and performed a meta-analysis to reveal the prognostic role of PLR in NSCLC.

## Results

### Study selection and characteristics

**Figure 1:**
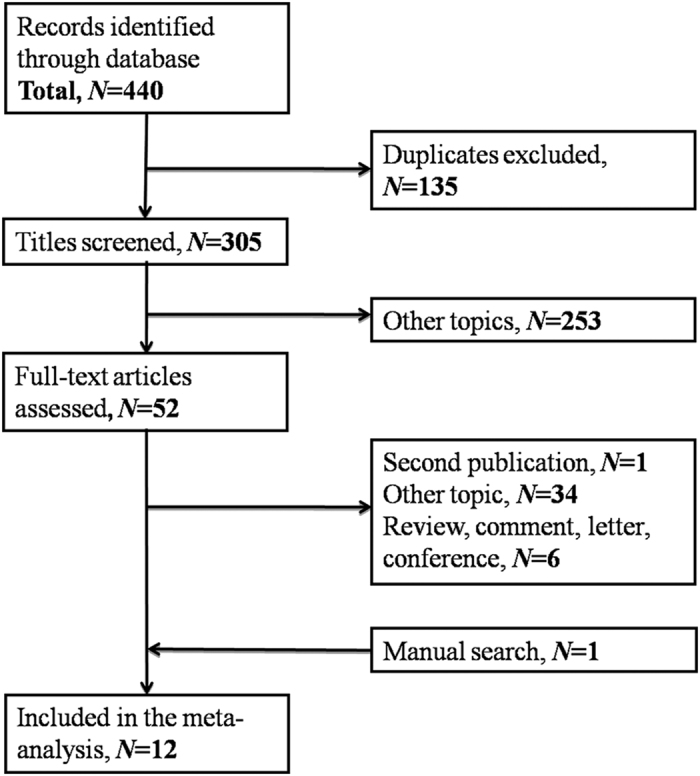
Flow diagram of studies selection procedure.

An electronic database search identified a total of 440 articles that met our criteria (Fig. 1). After removing duplicates, 305 citations were identified. Of these, 253 were excluded through an abstract review, leaving 52 articles. A full-text review of these articles resulted in the inclusion of 12^7^,^8^,^9^,^10^,^11^,^12^,^13^,^14^,^15^,^16^,^17^,^18^ studies in our meta-analysis.

The general features of the 12 eligible studies involving PLR are summarized in Table 1. These studies were published between 2012 and 2015, and included a total of 2,889 patients, ranging from 94 to 678 per study. Notably, five studies were conducted in China, three in Turkey, and one in Mexico, the UK, Japan and the USA. The cut-off values used for PLR in these studies were not consistent and ranged from 106 to 300. Seven studies used a PLR cut-off value of 150 or less, while five studies used a PLR greater than 150. HR and 95% CI were extracted directly in the original literature for nine studies, of which seven articles calculated HR and 95% CI by multivariate analysis, while the other two used univariate analysis. Although three articles did not provide HR and 95% CI, we were able to calculate these statistics from data provided in the article. The NOS scores of the studies ranged from 5 to 8, with a mean value of 7.

### Meta-analysis

**Figure 2:**
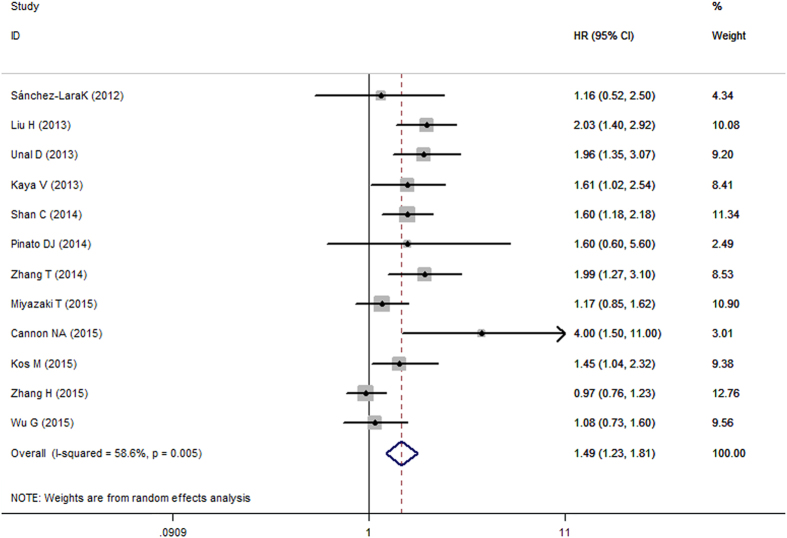
Forrest plot of hazard ratio (HR) for the association of PLR with overall survival (OS) in patients with NSCLC.

For the overall population, the pooled HR used to evaluate PLR on OS was 1.492 (95% CI: 1.231–1.807, P_heterogeneity_ = 0.005; Fig. 2), indicating that a high PLR was predictive of a poor prognosis in NSCLC patients. The main results of this meta-analysis are listed in Table 2. Subgroup analyses by ethnicity revealed that PLR was a negative predictor of OS for Asian (HR = 1.384, 95% CI: 1.067–1.795, P_heterogeneity_ = 0.003) and Caucasian populations (HR = 1.682, 95% CI: 1.348–2.099, P_heterogeneity_ = 0.432). Because the PLR cut-off values were different among the enrolled studies, we performed subgroup analyses based on different cut-off values and found that a high PLR was a negative predictor of OS both for a cut-off value of 150 or less (HR = 1.302, 95% CI: 1.028–1.648, P_heterogeneity_ = 0.029) and a PLR greater than 150 (HR = 1.831, 95% CI: 1.502–2.233, P_heterogeneity_ = 0.758). When considering differences in sample size, a high PLR was a poor prognostic marker for the sample sizes >200 (HR = 1.445, 95% CI: 1.074–1.945, P_heterogeneity_ = 0.003) and sample sizes ≤200 (HR = 1.546, 95% CI: 1.203–1.988, P_heterogeneity_ = 0.152). We also analyzed the significance of a high PLR with respect to OS for patients who received different treatments. Among five surgical and seven non-surgical studies, an elevated PLR was a uniformly poor predictor of OS (surgery: HR = 1.347, 95% CI: 1.012–1.793, P_heterogeneity_ = 0.022; non-surgery: HR = 1.624, 95% CI: 1.278–2.063, P_heterogeneity_ = 0.104). In addition, subgroup analyses showed that an elevated PLR predicted the prognosis for NSCLC, regardless of the HR estimation (reported vs. estimated) and NOS score (≥7 vs. <7, Table 2).

### Sensitivity analyses

We conducted meta-regression analysis to investigate the potential source of heterogeneity among studies of OS. The results demonstrated that ethnicity (*P* = 0.710), PLR cut-off value (*P* = 0.399), sample size (*P* = 0.983), treatment (*P* = 0.865), HR estimation (*P* = 0.660) and NOS score (*P* = 0.627) did not contribute to the source of heterogeneity for OS. Sensitivity analyses were used to examine whether individual studies influenced the results. When individual studies were removed one at a time from the above analyses, the corresponding pooled HRs were not markedly altered by any single study, indicating the robustness of the presented results.

### Publication bias

**Figure 3:**
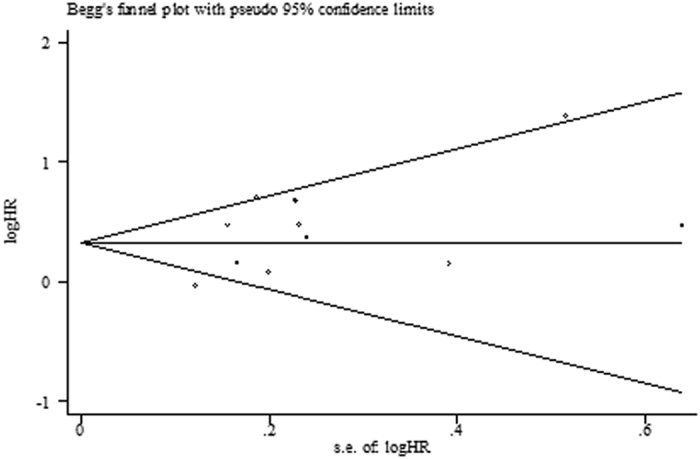
The funnel plot of the meta-analysis of the impact of PLR on overall survival (OS) in patients with NSCLC.

Begg’s funnel plot and Egger’s test were performed to evaluate the publication bias of the included studies. The funnel plot was symmetric for OS (*P* = 0.631, Fig. 3). Additionally, no indication of publication bias in terms of OS was found using Egger’s test (*P* = 0.089).

## Discussion

Inflammation is a hallmark of cancer^19^. Accumulated evidence shows that the systemic inflammatory response (SIR) is related to different stages of cancer progression, including its initiation, promotion, invasion and metastasis. A component of SIR, thrombocytosis is common in patients with solid tumors^20^,^21^. Through their secretion of VEGF, platelet-derived growth factor (PDGF), TGF-β and FGF, platelets can trigger angiogenesis, cell proliferation and migration^22^,^23^,^24^. Platelets not only augment the growth of primary tumors but also support cancer cells in their evasion of the immune system and extravasation to second sites. Nieswandt *et al*. have reported that platelets might protect cancer cells from natural killer-mediated lysis to facilitate metastasis^25^. Furthermore, Labelle *et al*. found that platelet-derived TGF-β promotes metastasis by activating the Smad and NF-kB pathways^26^. Based on these results, the prognostic value of thrombocytosis in various cancer patients was investigated^27^. Lymphocytes play an important role in the cell-mediated anti-tumor immune response. In colorectal and ovarian cancers, patients with a higher number of tumor-infiltrating lymphocytes (TILs) exhibit improved survival^28^,^29^. Kawai *et al*. reported that tumor-infiltrating CD8 + T cells in cancer nests have a positive effect on prognosis in cases of NSCLC^30^. Hence, platelets and lymphocytes might play a critical role in tumor progression. Because it is based on platelet and lymphocyte counts, the PLR could aid in predicting the clinical outcomes of cancer patients. Many studies have investigated the prognostic value of the PLR for various forms of cancer. However, the prognostic value of the PLR remains controversial for many ailments, including NSCLC. The aim of the current study was therefore to evaluate the prognostic value of the PLR for cases of NSCLC. To our knowledge, this is the first meta-analysis to investigate the association between the PLR and the prognosis of NSCLC patients.

By integrating the results of independent studies, meta-analysis provides a useful tool for the detection of effects that may be missed by individual studies. In this meta-analysis, we included 12 eligible studies of a total of 2,889 patients to clarify the prognostic value of PLR in NSCLC. The combined results demonstrated that a high PLR was associated with a poor OS (HR: 1.492, 95% CI: 1.231–1.807, *P* < 0.001) in NSCLC. In addition, the significance of this association was not changed in a sensitivity analysis that removed single studies. Subgroup analyses showed that the trend of a worse OS with a higher PLR was present in both Asian and Caucasian patients. We performed subgroup analyses based on PLR cut-off values and found that patients with a low PLR had a better OS compared to those with an elevated PLR, regardless of the PLR cut-off values. When analyzed by sample size, similarly significant results were found for both large and small studies. Subgroup analysis stratified by treatment (i.e., surgical or non-surgical), HR estimation (i.e., reported or estimated) and NOS (i.e., NOS ≥ 7 or < 7) also revealed that the PLR had a negative effect on OS. Our data help to clarify the results of individual studies and to identify NSCLC patients at high risk for whom adjuvant therapy might be necessary.

There is significant heterogeneity among the studies included in this meta-analysis. To explore the possible causes of heterogeneity, we performed subgroup analyses according to ethnicity, cut-off value, sample size, treatment, HR estimation and NOS. The results revealed that the subgroups representing Caucasian ethnicity, a cut-off value of PLR > 150, a sample size ≤200, non-surgical treatment, estimated HR and NOS score <7 had decreased heterogeneity. However, we could not rule out the possibility that heterogeneity was caused by these factors. For example, for PLR cut-off value, there was no universal threshold that defined a higher PLR in cancer patients. Different methods were used to calculate the PLR cut-off value in this meta-analysis, in which six studies set the cut-off value using a receiver operating curve (ROC), three by taking the median PLR, and three by using values reported in previously published literature. In addition, many factors could explain the heterogeneity observed among these studies, such as the clinical features of the enrolled patients, the method used to determine the laboratory index (such as platelet and lymphocyte counts), the pathological stage difference, or histology type.

Several limitations of this study must be carefully considered. First, significant heterogeneity was demonstrated in our results due to confounding factors such as the clinical features of the patients, ethnicity, sample size, treatment, HR estimation and PLR cut-off value. However, subgroup, meta-regression and sensitivity analyses revealed that none of the aforementioned confounders completely explained the observed heterogeneity. Thus, we propose that the genotypic diversity of NSCLC and the combined effect of the aforementioned confounders contributed to heterogeneity. Second, the HR of some studies was not provided, and we had to calculate the value from the data. There might be minor differences between the exact HR and the data extrapolated according to Tierney’s method. Third, although we did not impose a language limitation, only studies in English and Chinese were enrolled in the meta-analysis. Finally, studies lacking sufficient data were also excluded from the meta-analysis. Furthermore, due to negative or null results, many studies could not be published; these studies were also omitted from the present analysis. All of these factors might contribute to the heterogeneity observed in this study.

In conclusion, a meta-analysis of published studies revealed that PLR is an unfavorable predictor of prognosis in patients with NSCLC, which could be useful for stratifying NSCLC patients and in determining individual treatment plans. In addition, as a routine test, the PLR was easy to obtain and, importantly, does not incur additional costs. Thus, PLR is a promising biomarker for use in clinical management. However, due to the heterogeneity and limitations uncovered in the present study, the results of our meta-analysis may be estimations. Future larger-scale prospective and standard investigations should be conducted to confirm our results.

## Materials and Methods

### Search strategy

We conducted a comprehensive of the PubMed, Web of Science, and Embase databases to identify studies for inclusion in the present meta-analysis (last search updated to December 1, 2015). The following terms and combinations were used to identify studies: “lung cancer”, “lung carcinoma”, “lung neoplasm”, “lung malignancy”, “platelet”, “lymphocyte” and “ratio”. To ensure that no studies were overlooked, the reference lists of relevant articles and potential related articles were manually searched to identify additional studies.

### Date extraction and quality assessment

Two independent investigators (H.Z. and L.W.G.) read the titles and abstracts of all potential articles. Articles that could not be categorized based on title and abstract alone were retrieved for full-text review. The articles were read independently and checked for the inclusion criteria used in this study. For each study, the following data were collected: first author’s name, publication date, country of origin, ethnicity, number of patients, stage, treatment, cut-off value, follow-up, and hazard ratio (HR) of PLR for OS. Disagreement in the data extraction phase was settled together by the two reviewers. The Newcastle-Ottawa Quality Assessment Scale (NOS) was used to assess the quality of each study by two independent investigators (H.Z. and L.W.G.). The NOS consists of three parts: selection (four points), comparability (two points), and outcome assessment (three points). NOS scores ≥7 are considered to indicate high-quality studies.

### Inclusion and exclusion criteria

The inclusion criteria for this study were as follows: (1) patients with NSCLC in the studies were histo-pathologically confirmed; (2) the association of PLR with OS was investigated; and (3) the study reported sufficient data to estimate the HR at a 95% confidence interval (CI). The exclusion criteria were as follows: (1) reviews, letters, case reports, abstracts, conferences, or expert opinions; (2) studies in which necessary data were not provided; and (3) non-human research. We avoided the duplication of data by examining the names of all authors and medical centers involved for each article. When multiple publications using the same study population were identified or when the study populations overlapped, only the most complete and/or recently published was included.

### Statistical Analysis

The impact of PLR on OS was measured by the combined HRs and their 95% CIs extracted from each eligible study. The HR and its 95% CI in each eligible study were directly extracted from the report or were indirectly estimated by methods described by Tierney^31^. The Cochrane Q test and I^2^ statistic were performed to assess the heterogeneity of the pooled results. If *P* < 0.1 and/or I^2^ > 50%, indicating the presence of heterogeneity, the random-effects model (i.e., the DerSimonian-Laird method) was used to calculate the pooled HRs^32^. In other cases, the fixed-effects model (i.e., the Mantel-Haenszel method) was used^33^. The robustness of the pooled results was confirmed by a sensitivity analysis in which the data of an individual study was removed each time. Begg’s funnel plot and the Egger’s linear regression tests were conducted to identify the possibility of publication bias; *P* < 0.05 was considered significant. All statistical analyses were performed with Stata software version 11.0 (Stata Corporation, College Station, Texas, USA), and all P values were two-sided.

## Additional Information

**How to cite this article**: Zhang, H. *et al.* Prognostic value of platelet to lymphocyte ratio in non-small cell lung cancer: a systematic review and meta-analysis. *Sci. Rep.* **6**, 22618; doi: 10.1038/srep22618 (2016).

## Figures and Tables

**Table 1:** Baseline characteristics of the twelve studies included in the meta-analysis.

Study	Year	Country	Ethnicity	Number	Stage	Treatment	Cut-off	Follow-up (m)	HR estimate	NOS
Sánchez-LaraK	2012	Mexico	Caucasian	119	III/IV	Chemotherapy	150	6	Reported	6
Liu H	2013	China	Asian	210	III/IV	Chemotherapy	152.6	18.6	Reported	7
Unal D	2013	Turkey	Caucasian	94	II/III	Chemo-radiotherapy	194	NR	Estimated	5
Kaya V	2013	Turkey	Caucasian	156	III/IV	NR	150	12.5	Estimated	6
Shan C	2014	China	Asian	255	I-III	Surgery	130	36	Reported	8
Pinato DJ	2014	UK	Caucasian	220	I-IIIA	Surgery	300	13	Reported	7
Zhang T	2014	China	Asian	400	I/II	Surgery	171	46	Reported	8
Miyazaki T	2015	Japan	Asian	97	I-IV	Surgery	118	NR	Estimated	6
Cannon NA	2015	USA	Caucasian	149	I	Radiotherapy	146	17	Reported	8
Kos M	2015	Turkey	Caucasian	145	I-IV	NR	198.2	33	Reported	8
Zhang H	2015	China	Asian	678	I-IIIA	Surgery	106	44	Reported	8
Wu G	2015	China	Asian	366	III/IV	Chemotherapy	119.5	30	Reported	7

NR: not reported; m: month; HR: hazard ratio.

**Table 2:** Subgroup analysis of pooled Hazard ratios (HRs) reflecting the association between PLR and OS in NSCLC patients.

Subgroup	No. of studies	No. of patients	HR	95% CI	*P* value	Model	Heterogeneity	
							I^2^ (%)	P_Heterogeneity_
Ethnicity								
Asian	6	2,006	1.384	1.067–1.795	0.014	Random	72.3	0.003
Caucasian	6	883	1.682	1.348–2.099	<0.001	Fixed	0	0.432
Cut-off value								
≤150	7	1,820	1.302	1.028–1.648	0.029	Random	57.3	0.029
>150	5	1,069	1.831	1.502–2.233	<0.001	Fixed	0	0.758
Sample size								
≤200	6	760	1.546	1.203–1.988	0.001	Fixed	32.2	0.152
>200	6	2,129	1.445	1.074–1.945	0.015	Random	71.7	0.003
Treatment								
Surgery	5	1,650	1.347	1.012–1.793	0.041	Random	65.1	0.022
Non-surgery	7	1,239	1.624	1.278–2.063	<0.001	Fixed	40.5	0.104
HR estimation								
Reported	9	2,542	1.493	1.165–1.915	0.002	Random	64.3	0.004
Estimated	3	347	1.511	1.101–2.075	0.011	Fixed	48.6	0.143
NOS score								
≥7	7	2,423	1.526	1.170–1.991	0.002	Random	68.5	0.002
<7	5	466	1.462	1.121–1.906	0.005	Fixed	28.9	0.239

No.: number; HR: hazard ratio; CI: confidence interval.
